# Clinical and genomic characterization of corpus callosum abnormalities (CCA) in 107 Tunisian patients using a stepwise diagnostic approach

**DOI:** 10.3389/fgene.2026.1815268

**Published:** 2026-09-14

**Authors:** Bochra Khadija, Hamza Hadj Abdallah, Wafa Slimani, Ayda Bennour, Sarra Dimassi, Najla Soyah, Amira Benzarti, Khouloud Rjiba, Wafa Dahleb, Molka Kammoun, Hanen Hannechi, Hela Ben Khelifa, Neziha Gouider Khouja, Lamia Boughamoura, Ichrak Kraoua, Chahnaz Triki, Amel Tej, Jihen Mathlouthi, Aida Guith, Saoussen Abroug, Raoudha Kebaili, Mohamed Ali Bouaziz, Habib Soua, Essia Sboui, Samir Hadded, Randa Ziadi, Kamel Monastiri, Hayet Ben Hamida, Monji Ghanmi, Sayda Hassayoun, Mohamed Tahar Sfar, Ali Saad, Christel Depienne, Soumaya Mougou-Zerelli

**Affiliations:** 1 Common Service Unit for Research in Genetics, Faculty of Medicine of Sousse, University of Sousse, Sousse, Tunisia; 2 Laboratory of Cytogenetics, Molecular Genetics and Human Reproductive Biology, CHU Farhat Hached, Sousse, Tunisia; 3 Pediatrics Department, CHU Farhat Hached Sousse, Sousse, Tunisia; 4 Service of Neuropediatrics, National Institute of Neurology of Tunis, Tunis, Tunisia; 5 Neuropediatrics Department, Hedi Chaker Hospital, Sfax, Tunisia; 6 Service of Neonatology, CHU Farhat Hached Sousse, Faculty of Medicine of Sousse, University of Sousse, Sousse, Tunisia; 7 Pediatrics Department, Sahloul University Hospital Sousse, Sousse, Tunisia; 8 Private Practitioner, Tunis, Tunisia; 9 Neonatology Department, Tahar Sfar Hospital Mahdia, Mahdia, Tunisia; 10 Pediatrics Department, Ibn El Jazzar Hospital, Faculty of Medicine of Sousse, University of Sousse, Kairouan, Tunisia; 11 Pediatrics Department, Bechir Hamza Children Hospital, Faculty of Medicine of Tunis, University of Tunis El Manar, Tunis, Tunisia; 12 Pediatrics Department, Fattouma Bourguiba University Hospital, University of Monastir, Monastir, Tunisia; 13 Neonatal Medicine Department, Fattouma Bourguiba University Hospital, University of Monastir, Monastir, Tunisia; 14 Neonatology Department, Farhat Hached University Hospital, Faculty of Medicine of Sousse, University of Sousse, Sousse, Tunisia; 15 Pediatric Department, Sahloul Hospital, Sousse, Tunisia; 16 Pediatrics Department, Tahar Sfar Hospital, Faculty of Medicine of Monastir, University of Monastir, Mahdia, Tunisia; 17 Institute of Human Genetics, University Hospital Essen, University of Duisburg-Essen, Essen, Germany

**Keywords:** copy-number variations (CNVs), corpus callosum malformations, neurodevelopmental genomics, rare genetic disorders, stepwise approach

## Abstract

**Background:**

Corpus callosum abnormalities (CCA) represent a heterogeneous group of neurodevelopmental disorders resulting from disturbances in midline patterning, neuronal migration, and axonal guidance. Their genetic architecture includes chromosomal abnormalities, pathogenic copy-number variations (CNVs), and single-gene disorders; however, genotype–phenotype correlations remain incompletely defined, particularly in underrepresented populations.

**Methods:**

We investigated the clinical and genomic characteristics of 107 Tunisian patients with radiologically confirmed CCA recruited over a 15-year period. A sequential genomic strategy was applied, including conventional karyotyping in all patients, fluorescence *in situ* hybridization (FISH) and multiplex ligation-dependent probe amplification (MLPA) when clinically indicated, chromosomal microarray analysis (array-CGH) in 54 selected patients, and whole-exome sequencing (WES) in six unresolved cases. CNVs were interpreted according to ACMG/ClinGen recommendations, and sequence variants were classified according to ACMG/AMP criteria.

**Results:**

Most patients presented syndromic forms of CCA (95.3%) associated with additional cerebral and/or extracerebral manifestations. Clinically relevant genomic abnormalities supporting a molecular diagnosis were identified in 11/107 patients (10.3%). Conventional karyotyping identified chromosomal abnormalities in three patients (2.8%), while array-CGH detected pathogenic or likely pathogenic CNVs in ten of 54 tested patients (18.5%). These CNVs involved genomic regions previously implicated in neurodevelopmental disorders and CCA, including 14q12, 4p16.3–p16.1, 1q43–q44, 1p32.3p31.3 –p32, 5p14.3, 5q35, 16q23.1–q24.3, and 18pterp11.1. WES identified rare variants in *WLS*, *ERCC8*, and *VPS39*, while a *MID1* variant was classified as likely benign. No sequence variant met ACMG/AMP criteria for pathogenicity, and none was considered diagnostic.

**Conclusion:**

This study provides a comprehensive characterization of CCA in a Tunisian referral cohort and highlights the major contribution of pathogenic genomic imbalances to the molecular diagnosis of these disorders. The findings emphasize the complementary role of cytogenetic and sequencing approaches while illustrating the challenges of interpreting rare sequence variants in patients with complex neurodevelopmental phenotypes. Larger cohorts with systzbematic genomic testing, segregation analyses, and functional validation will be required to further refine genotype–phenotype correlations in CCA.

## 1 Introduction

The corpus callosum is the largest commissural structure of the human brain and plays a fundamental role in interhemispheric communication by integrating sensory, cognitive, and motor information between the cerebral hemispheres. Its development is a highly coordinated embryological process involving neuronal proliferation, migration, axon guidance, midline patterning, and commissural crossing. Disruption of any of these processes results in corpus callosum abnormalities (CCA), a heterogeneous spectrum that includes complete agenesis, partial agenesis, hypoplasia, and dysgenesis, with highly variable clinical manifestations ranging from isolated findings to complex neurodevelopmental disorders (De León Reyes et al., 2020; Mowat et al., 2014).

Corpus callosum abnormalities are among the most frequently reported congenital anomalies of the central nervous system. Population-based studies estimate their prevalence at approximately 2–3 per 10,000 live births, although reported frequencies vary according to study design, prenatal diagnosis, and ascertainment strategy (Glass et al., 2008; Edwards et al., 2014). Clinical outcomes are highly heterogeneous, ranging from normal neurodevelopment in isolated cases to severe developmental delay, intellectual disability, epilepsy, and multiple congenital anomalies in syndromic forms.

The formation of the corpus callosum depends on tightly regulated molecular pathways controlling commissural axon guidance and midline development. Several genes and signaling pathways have been implicated in this process, including the Netrin-1/DCC, L1CAM, ARX, ZEB2, Wnt, and Semaphorin–Plexin–Neuropilin pathways, which regulate neuronal migration, axonal navigation, and interactions with specialized midline glial structures required for successful commissural crossing (De León Reyes et al., 2020; Ku and Torii, 2020).

The genetic architecture of CCA is highly heterogeneous and includes chromosomal abnormalities, pathogenic copy-number variations (CNVs), and single-gene disorders. The implementation of chromosomal microarray analysis and next-generation sequencing has substantially improved the molecular diagnosis of affected individuals, particularly in syndromic cases. To date, the genetic cause is identifiable for 30%–45% of individuals with CCA with around 10% of them having chromosomal anomalies and the remaining 20%–35% with recognizable genetic syndromes with a mutation affecting a single gene (Edwards et al., 2014; Jouan et al., 2016).

However, reported diagnostic yields vary considerably according to cohort composition, imaging phenotype, and the genomic testing strategy employed, making comparisons between studies challenging (Edwards et al., 2014; Bayram et al., 2020).

Several studies from Tunisia have described chromosomal and monogenic causes of corpus callosum abnormalities and related neurodevelopmental disorders, including recurrent microdeletion syndromes involving chromosomal regions such as 14q12, 1q43–q44, and 1p36, as well as pathogenic variants in genes implicated in brain (Ouertani et al., 2009; Kerkeni et al., 2026). Nevertheless, most published data are limited to isolated case reports or small case series. Comprehensive studies integrating clinical evaluation with sequential cytogenetic, chromosomal microarray, and exome sequencing analyses in a large Tunisian cohort remain scarce. Such studies are essential to improve molecular diagnosis, refine genotype–phenotype correlations, and better characterize the genetic heterogeneity of corpus callosum abnormalities in this population.

In the present study, we investigated a cohort of 107 Tunisian patients with radiologically confirmed corpus callosum abnormalities using a stepwise genomic approach combining conventional karyotyping, fluorescence *in situ* hybridization (FISH), chromosomal microarray analysis (array-CGH), and whole-exome sequencing in selected unresolved cases. Our objectives were to (i) describe the clinical and neuroradiological spectrum of corpus callosum abnormalities, (ii) report the diagnostic findings obtained at each stage of the sequential genomic workflow, and (iii) explore the contribution of structural and sequence variants to the molecular characterization of selected patients.

## 2 Materials and methods

### 2.1 Ethics statement

The study was approved by the Ethics Committee of Farhat Hached University Hospital (Approval No. CER 17–2022). The cohort included patients referred to the Department of Genetics between January 2010 and December 2025. Patients recruited before 2022 underwent genetic investigations as part of routine clinical care. Following ethics approval in 2022, written informed consent was obtained from patients or their legal guardians for the use of anonymized clinical and genetic data for research purposes. Patients recruited after ethics approval provided written informed consent prospectively, in accordance with institutional and national regulations.

### 2.2 Patient selection

This cross-sectional observational study included 107 patients from 106 unrelated families, including one pair of affected siblings with corpus callosum abnormalities referred to the Department of Genetics, Farhat Hached University Hospital (Sousse, Tunisia), between January 2010 and December 2025. Patients were referred for genetic evaluation because of developmental delay, intellectual disability, epilepsy, dysmorphic features, congenital anomalies, or brain malformations.

### 2.3 Clinical and neuroradiological assessment

Clinical information was collected from medical records, including demographic characteristics, family history, parental consanguinity, neurological manifestations, dysmorphic features, associated congenital anomalies, and previous genetic investigations.

Brain MRI examinations had been performed by the referring doctors as part of routine clinical care before referral for genetic evaluation. MRI reports were reviewed, and corpus callosum abnormalities were classified according to the neuroradiological diagnosis reported by the referring radiologists as complete agenesis, partial agenesis, hypoplasia, or unspecified corpus callosum abnormality when the available reports did not allow further subclassification.

Patients were classified as having isolated CCA or syndromic CCA depending on the presence of additional cerebral or systemic abnormalities. Because MRI examinations were acquired over a 15-year period in different institutions using non-standardized imaging protocols, centralized image review and assessment of inter-observer agreement were not feasible. This represents a limitation of the study and was considered during interpretation of the findings.

### 2.4 Genetic analyses

#### 2.4.1 Conventional karyotyping

Conventional cytogenetic analysis was performed for all 107 patients using peripheral blood lymphocyte cultures according to standard procedures. Peripheral blood samples were collected in lithium heparin tubes and cultured for 72 h in RPMI 1640 medium supplemented with fetal bovine serum, L-glutamine, antibiotics, and phytohemagglutinin (PHA) to stimulate T-lymphocyte proliferation. Cell cultures were maintained at 37 °C in a humidified atmosphere containing 5% CO_2_. Metaphase arrest was achieved by colchicine treatment, followed by hypotonic treatment with 0.075 M KCl and fixation using methanol/acetic acid (3:1).

Metaphase spreads were prepared under controlled temperature and humidity conditions and subjected to R-banding after thermal denaturation in Earle’s buffer. Chromosomes were stained with Giemsa and analyzed using an Axioskop Zeiss® microscope coupled to Cytovision® software (Applied Imaging).

For each patient, 15–25 metaphases were examined at a resolution of approximately 450–550 bands. Karyotypes were interpreted according to the International System for Human Cytogenomic Nomenclature (ISCN 2020).

#### 2.4.2 Fluorescence *In Situ* Hybridization (FISH)

Fluorescence *in situ* hybridization (FISH) was performed in selected patients for the confirmation and characterization of large chromosomal rearrangements, subtelomeric abnormalities, marker chromosomes, and selected clinically relevant CNVs within the resolution limits of the technique when probes are available. When parental DNA samples were available, FISH was also used to investigate the inheritance of selected chromosomal abnormalities. Commercial locus-specific probes were used according to the manufacturer’s recommendations.

Metaphase spreads were prepared from peripheral blood lymphocytes and pretreated using standard protocols including SSC washes, paraformaldehyde fixation, pepsin digestion, and ethanol dehydration. Probe and chromosomal DNA co-denaturation was performed at 75 °C for 7 min, followed by overnight hybridization at 37 °C using the Thermobrite® system (Abbott Molecular). Post-hybridization washes were carried out in 2× SSC/NP-40 buffer under stringent conditions. Chromosomes were counterstained with DAPI. Hybridization signals were analyzed using an Axioskop Zeiss® epifluorescence microscope equipped with appropriate fluorochrome filters and Cytovision® image analysis software (Applied Imaging). FISH analysis provided an estimated resolution of approximately 1–3 Mb depending on probe design and genomic region.

#### 2.4.3 Array comparative genomic hybridization (array CGH)

Chromosomal microarray analysis (array-CGH) was performed in 54 selected patients using the Agilent SurePrint G3 Human CGH Microarray 4 × 44K platform (Agilent Technologies) according to the manufacturer’s protocol. The platform has an average of approximately 44,000 oligonucleotide probes with an average genomic resolution of ∼43–75 kb corresponding to an effective detection resolution of approximately 130–225 kb, depending on local probe density.

Genomic DNA was extracted from peripheral blood using the FlexiGene DNA Kit (Qiagen, Germany) according to the manufacturer’s instructions. DNA concentration and purity were assessed using NanoDrop spectrophotometry, and samples with A260/A280 ratios between 1.8 and 2.0 were considered suitable for analysis. After labeling and purification, equal quantities of patient and control DNA were co-hybridized onto the microarray slides in the presence of Cot-1 DNA to suppress repetitive sequence hybridization.

Hybridization was performed for 24 h at 65 °C in a rotating hybridization oven. Following post-hybridization washes, arrays were scanned. Raw data were analyzed using Agilent DNA Analytics software version 4.0. CNVs were identified using the ADM-2 algorithm with the manufacturer’s recommended quality thresholds. copy-number variations (CNVs) were considered significant when at least three contiguous probes showed concordant deviation in fluorescence ratio. Thresholds for CNV calling were defined as log_2_ ratio > +0.58 for gains and < −0.75 for losses. Genomic coordinates were reported according to the hg18 human reference genome. CNVs were classified following the American College of Medical Genetics and Genomics (ACMG) and Clinical Genome Resource (ClinGen) technical standards for constitutional copy-number variation interpretation (Riggs et al., 2020).

Interpretation incorporated CNV size, genomic content, overlap with known genomic disorders, dosage-sensitive genes, inheritance (when parental samples were available), and evidence from ClinGen, ClinVar, DECIPHER, OMIM, the Database of Genomic Variants (DGV), and the published literature. CNV interpretation workflow used in this study is shown in Figure 1.

**FIGURE 1:**
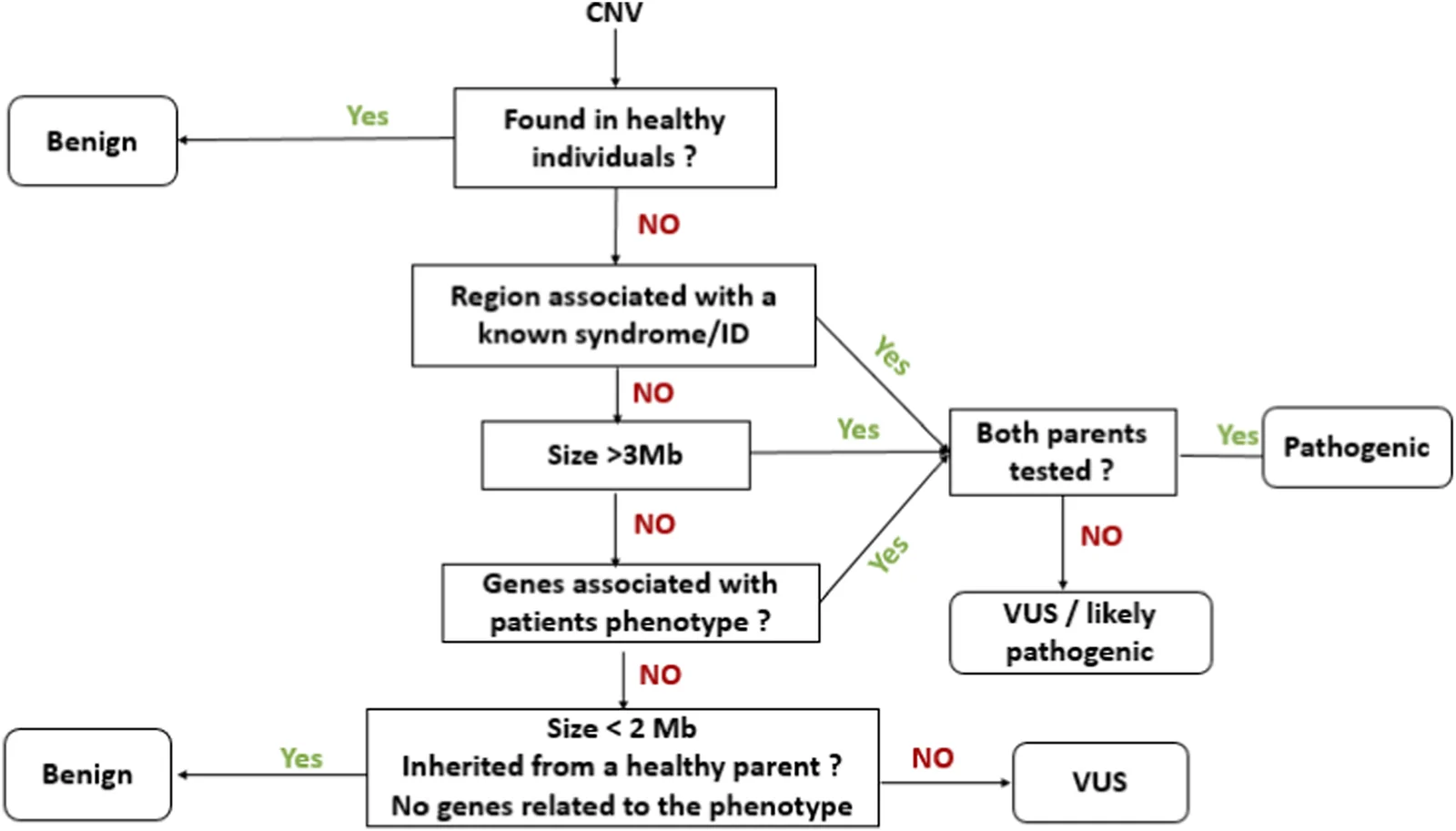
Flowchart *for CNV classification according to clinical significance.* This flowchart illustrates the workflow used to classify copy-number variations (CNVs) detected by array CGH. CNVs are first evaluated for their presence in healthy individuals and association with known syndromes or intellectual disability (ID) regions. Additional criteria, including CNV size, gene content, inheritance pattern, and parental testing results, are used to determine their clinical relevance. CNVs are classified as benign, variant of uncertain significance (VUS), likely pathogenic, or pathogenic according to these parameters.

#### 2.4.4 Multiplex ligation-dependent probe amplification (MLPA)

Multiplex ligation-dependent probe amplification (MLPA) was used to detect targeted copy number changes in specific chromosomal regions. Analyses were performed using the SALSA MLPA P064 kit. Briefly, 150 ng of genomic DNA was denatured, hybridized with probe mixes, ligated, and amplified by PCR. Amplification products were analyzed by capillary electrophoresis on an ABI PRISM 310 Genetic Analyzer. Data interpretation was carried out using GeneMarker® and Coffalyser® software to identify deletions or duplications. MLPA was performed in patients (only in-patient P10) with suspected targeted copy-number alterations when array-CGH was unavailable or when confirmation of specific loci was clinically indicated.

#### 2.4.5 Whole-exome sequencing

Whole-exome sequencing (WES) was performed in six selected patients whose clinical phenotype remained incompletely explained after chromosomal microarray analysis. This approach aimed to identify additional pathogenic single-nucleotide variants or small insertions/deletions acting independently or in combination with CNVs, such as recessive variants unmasked by deletions, compound heterozygosity, or additional sequence variants potentially contributing to phenotypic variability.

Exome libraries were prepared using the Twist EF Library Preparation Kit 2.0 and the Twist Comprehensive Exome Panel (Twist Bioscience, South San Francisco, CA, USA). Paired-end sequencing (2 × 150 bp) was performed on an Illumina NovaSeq X Plus platform. Sequencing quality metrics included a mean target coverage of approximately 100×, with more than 95% of target bases covered at ≥20× and Q30 scores exceeding 90%.

#### 2.4.6 Bioinformatic analysis and variant interpretation

Sequence reads were aligned to the GRCh37/hg19 human reference genome and variants were called using the DRAGEN Bio-IT Platform (Illumina) on the Illumina NovaSeq X Plus platform. Variant annotation was performed using ANNOVAR. Variant prioritization incorporated phenotype-driven analysis and inheritance models, including *de novo*, autosomal dominant, autosomal recessive, X-linked, and compound heterozygous inheritance. Variants were interpreted using Franklin by Genoox (accessed October 2023), ClinVar, gnomAD, OMIM, and DECIPHER. Variants with a minor allele frequency ≥1% in population databases were excluded unless previously associated with a Mendelian disorder or supported by strong clinical evidence. Sequencing quality metrics included a mean target coverage of approximately 100×, with more than 95% of target bases covered at ≥20× and Q30 scores exceeding 90%. Variant-level quality parameters, including sequencing depth and allelic depth, were reviewed during variant interpretation. For homozygous candidate variants identified in patients carrying CNVs, allele balance was assessed from alternative and reference read counts to confirm the reliability of homozygous genotype calls and exclude potential misinterpretation due to hemizygosity.

Sequence variants were classified according to the ACMG/AMP guidelines (Richards et al., 2015). For each variant, the ACMG evidence criteria applied (e.g., PM, PP, BS) were documented together with population frequency, available segregation data, and previously reported clinical evidence when available. Sanger sequencing confirmed candidate variants in patients P16-P17-P18, and segregation analysis was performed in available family members whenever possible. The ACMG/AMP criteria supporting each classification are provided in Table 1.

**TABLE 1:** Sequence variants identified by WES in four patients.

Patient	Sex/Age	Gene	Transcript (MANE)	HGVS cDNA	HGVS protein	Zygosity	ACMG evidence	Classification	gnomAD	SIFT	Polyohen	PHRED	Segregation	Sanger	ClinVar
P7	F 11 Y	MID1	NM_ 000,381.4	c.250G>A	p.Asp84Asn	Het	PM2, BP6, BS2	Likely benign	Absent	Uncertain (0.001)	Uncertain (0.9)	26.1	Maternal	No	SCV007595998
P16	F 7 Y	WLS	NM_024911.7	c.985C>T	p.Arg329Trp	Het	PM2	VUS	Absent	Uncertain (0.001)	Deleterious (Moderate) (1)	​	Parents pending	Yes	SCV007595994
P16	F 7 Y	WLS	NM_024911.7	c.1124C>A	p.Thr375Lys	Het	PM2	VUS	Absent	​	​	​	Parents pending	Yes	SCV007595995
P16	F 7 Y	ERCC8	NM_ 000,082.4	c.598T>A	p.Tyr200Asn	Homo	PM2, PM1	VUS	Absent	​	​	​	Parents pending	Yes	SCV007595996
P17	M 1 Y	VPS39	NM_015289.5	c.1648G>T	p.Val550Leu	Homo	PM2	VUS	Absent	​	​	​	Segregated	Yes	SCV007595997
P18	F 3 Y	VPS39	NM_015289.5	c.1648G>T	p.Val550Leu	Homo	PM2	VUS	Absent	​	​	​	Segregated	Yes	SCV007595997

Abbreviations: F, Female, M, Male, Het, Heterozygous, Homo, Homozygous, P, patient, Y, years.

## 3 Results

### 3.1 Clinical characteristics of the cohort

A total of 107 patients with radiologically confirmed corpus callosum abnormalities (CCA) were included in this study. MRI reports obtained from the referring hospitals confirmed the diagnosis in all patients. Based on the available neuroradiological descriptions, 25 patients (23.4%) presented complete agenesis of the corpus callosum, 11 (10.3%) partial agenesis, and 36 (33.6%) corpus callosum hypoplasia. In the remaining 31 patients (28.9%), MRI reports confirmed the presence of corpus callosum abnormalities but did not provide sufficient radiological detail to distinguish between complete agenesis, partial agenesis, or hypoplasia.

Among the 107 patients (53 males, 54 females), 102 (95.3%) presented syndromic forms associated with additional cerebral and/or extracerebral manifestations, whereas five patients (4.7%) had apparently isolated corpus callosum abnormalities. Because isolated CCA were observed in only five patients, formal comparative subgroup analyses between isolated and syndromic cases were not performed. Frequent associated brain abnormalities included hydrocephalus, holoprosencephaly, cerebellar hypoplasia, cortical malformations (polymicrogyria, nodular heterotopia, focal cortical dysplasia), septal defects, and posterior fossa malformations such as Dandy–Walker spectrum anomalies. Extracerebral anomalies mainly involved ocular, cardiac, and genital systems. The high proportion of syndromic cases reflects the hospital-based recruitment of patients referred to a tertiary genetics service rather than the true distribution of corpus callosum abnormalities in the general population. Consanguinity was documented in 40/107 (37%) patients. Representative dysmorphic features observed in selected patients are shown in Figure 2.

**FIGURE 2:**
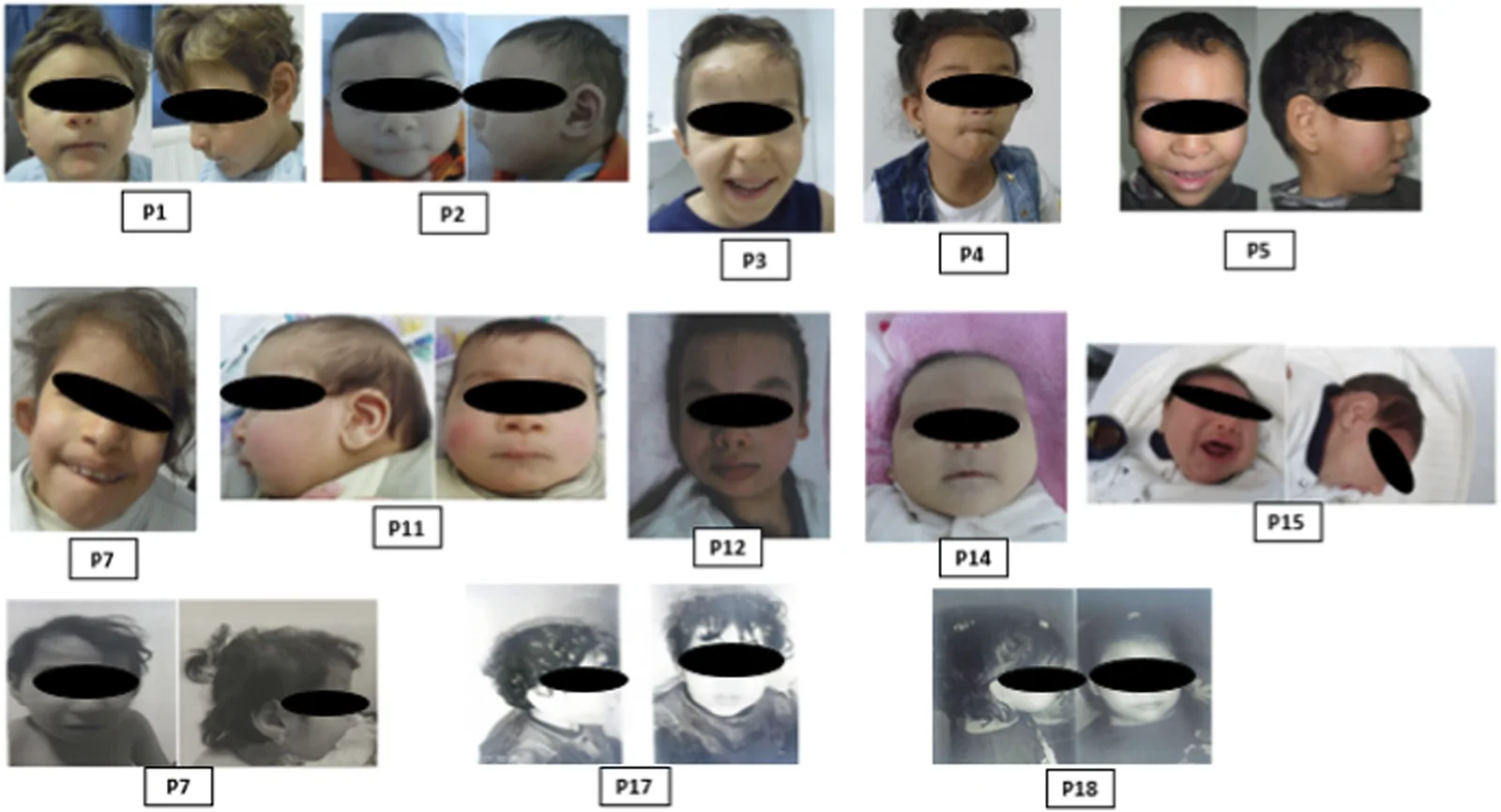
Presentation of patients with available photographs showing facial dysmorphic features associated with identified genetic anomalies in our cohort.

### 3.2 Stepwise cytogenetic and genomic investigations

A sequential genomic strategy was applied according to clinical indication and test availability. All patients underwent conventional karyotyping. FISH was performed when clinically indicated to confirm or further characterize chromosomal abnormalities. Array-CGH was performed in 54 patients with suspected genomic imbalances, and whole-exome sequencing (WES) was subsequently performed in six selected unresolved patients carrying CNVs and/or presenting parental consanguinity. The workflow followed in this study and the genomic landscape are shown in Figure 3.

**FIGURE 3:**
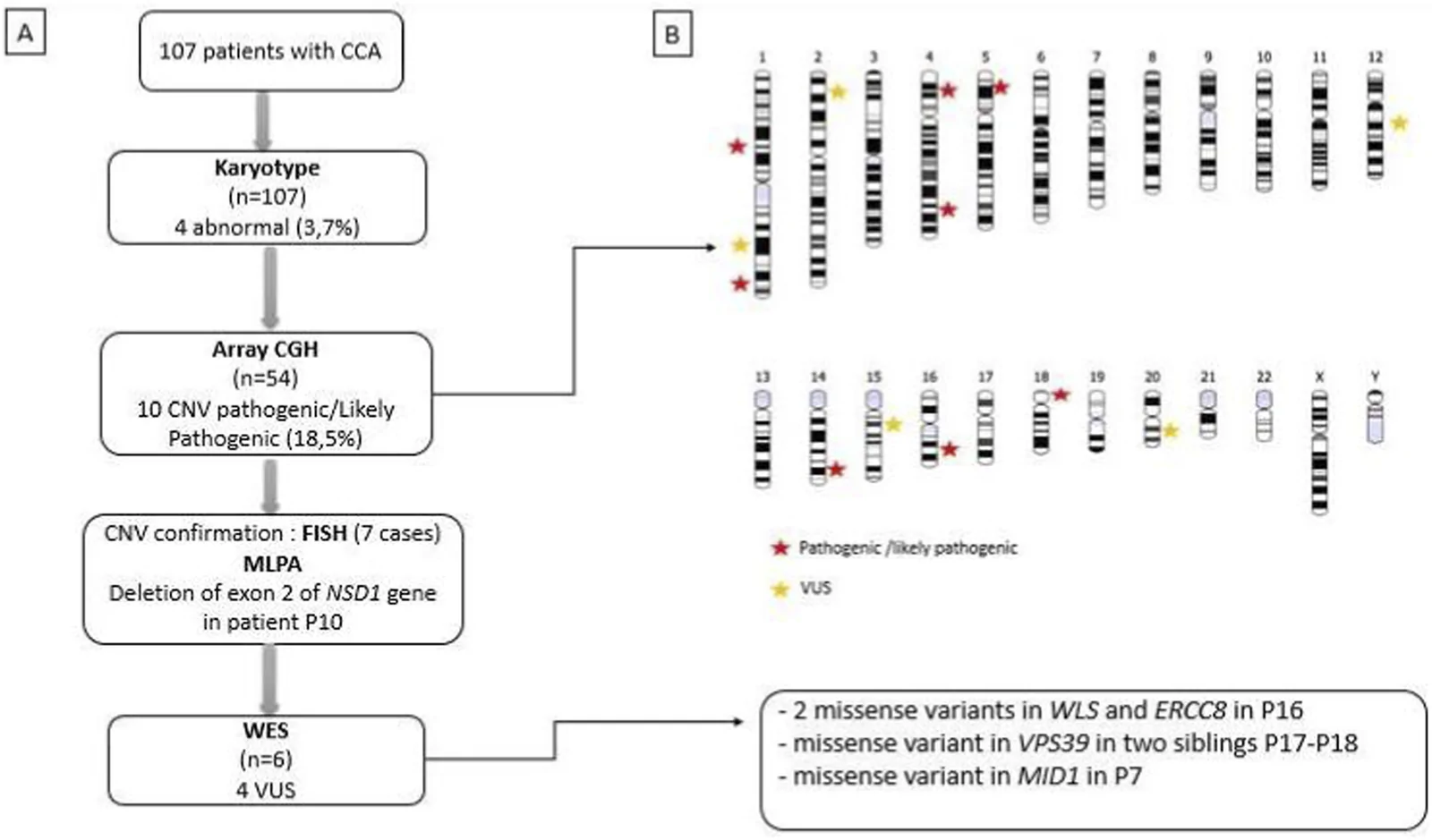
**(A)** Diagnostic workflow, **(B)** Genomic alterations identified.

### 3.3 Conventional and molecular cytogenetic analysis

Conventional karyotyping identified chromosomal abnormalities in three of the 107 patients (2.8%). These included deletions/duplications identified in P5, P11, and P14, which were subsequently characterized by array-CGH. Karyotype profiles with anomalies are presented in Figure 4. These abnormalities were subsequently characterized by array-CGH as pathogenic copy-number variations. FISH analysis was performed in 24 patients and confirmed or further characterized chromosomal abnormalities in seven cases 29.2% of tested patients. FISH profiles with anomalies are presented in Figure 5.

**FIGURE 4:**
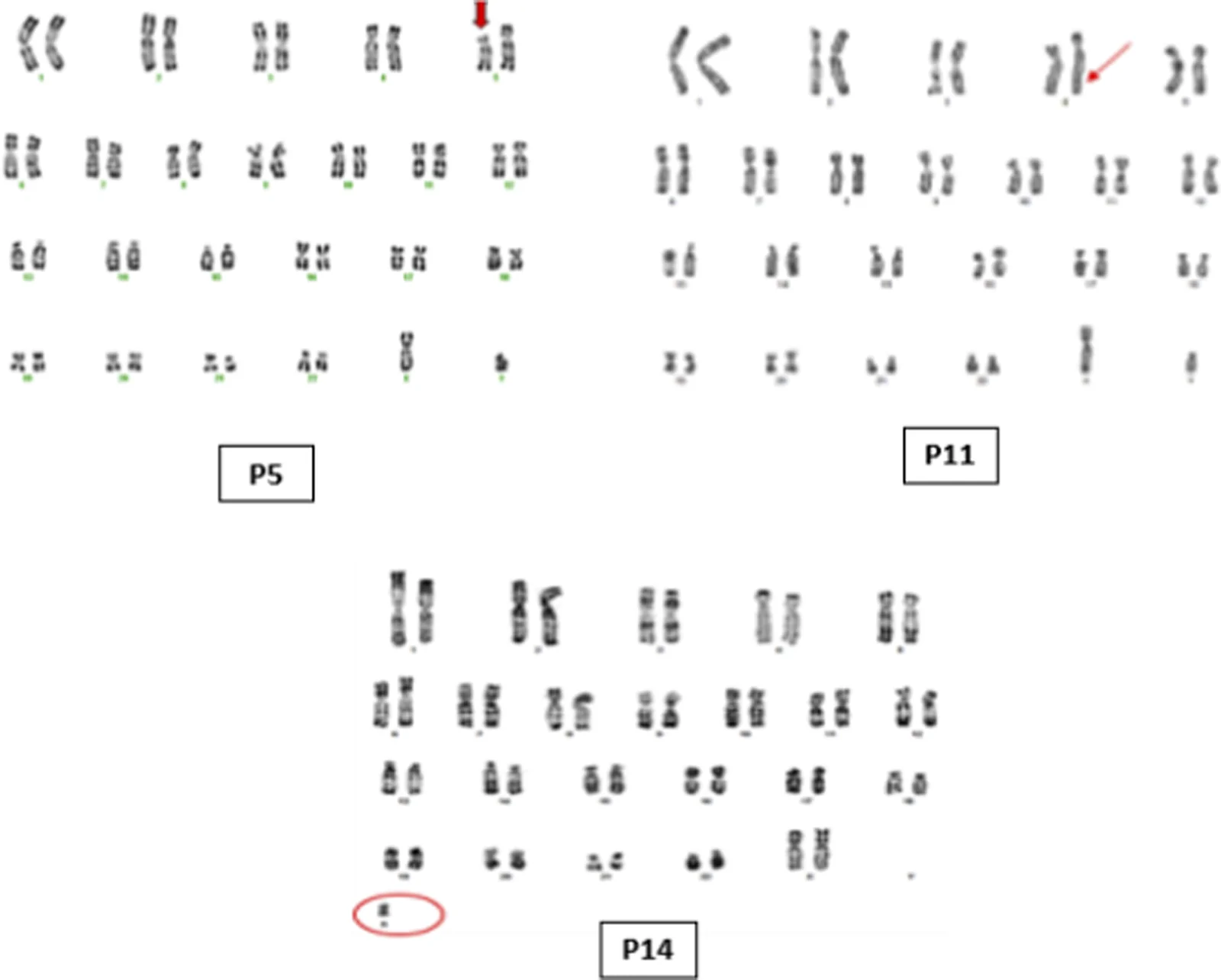
Standard karyotype results showing chromosomal abnormalities: P5 with 5p14.3 deletion (46,XY, del(5)(p14.3)), P11 with 4q31–q35 duplication (46,XX, dup(4)(q31q35)), and P14 with a chromosomal marker (A: chromosomal marker).

**FIGURE 5:**
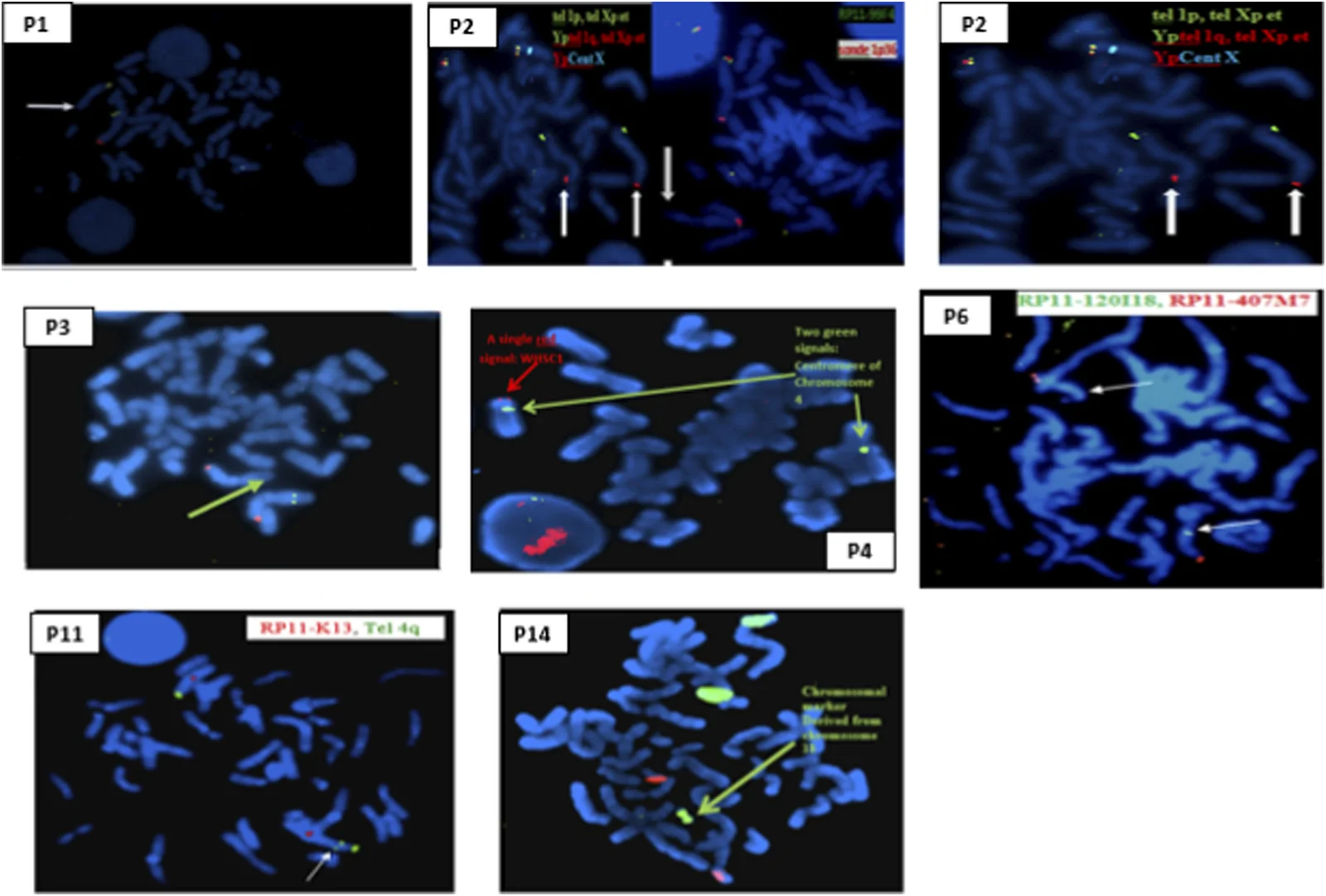
FISH (Fluorescence *In Situ* Hybridization) used to confirm CNVs detected by array CGH when corresponding probes are available. P1: Bluefish probe of telomere 1q: corresponding to the CEP170 gene and a control probe 1p36 absence of the second red signal confirming the terminal loss at 1q43q44. P2: confirmation of the interstitial deletion by performing FISH on the two q telomeres of the two homologous chromosomes 1. P3: Absence of hybridization of the RP11-714D6 probe (green arrow) on one of the two homologous chromosomes 1, confirming the deletion at 1p32.3p31.3. P4: Absence of hybridization of the WHSC1 probe (red arrow) on one of the two homologous chromosomes 4, confirming the deletion. P6: Absence of the hybridization signal on one of the two homologous chromosomes 14, confirming the presence of a microdeletion. P11: Presence of two green signals on one of the homologous chromosomes 4, confirming the duplication at this locus. P14: Triple green signal from the whole-chromosome painting (WHS) probe for chromosome 18 and two signals for chromosome 22.

Array-CGH was performed in 54 patients and identified 18 copy-number variations in 16 patients (29.6%). According to ACMG/ClinGen recommendations for CNV interpretation (Riggs et al., 2020), pathogenic or likely pathogenic CNVs were identified in 10 patients, corresponding to a diagnostic yield of 18.5% (10/54). The remaining CNVs were classified as variants of uncertain significance. Representative array-CGH profiles are shown in Figure 6. The 10 patients carrying pathogenic or likely pathogenic CNVs detected by array-CGH were P1, P2, P3, P4, P5, P6, P8, P9, P11, and P14 (Table 3).

**FIGURE 6:**
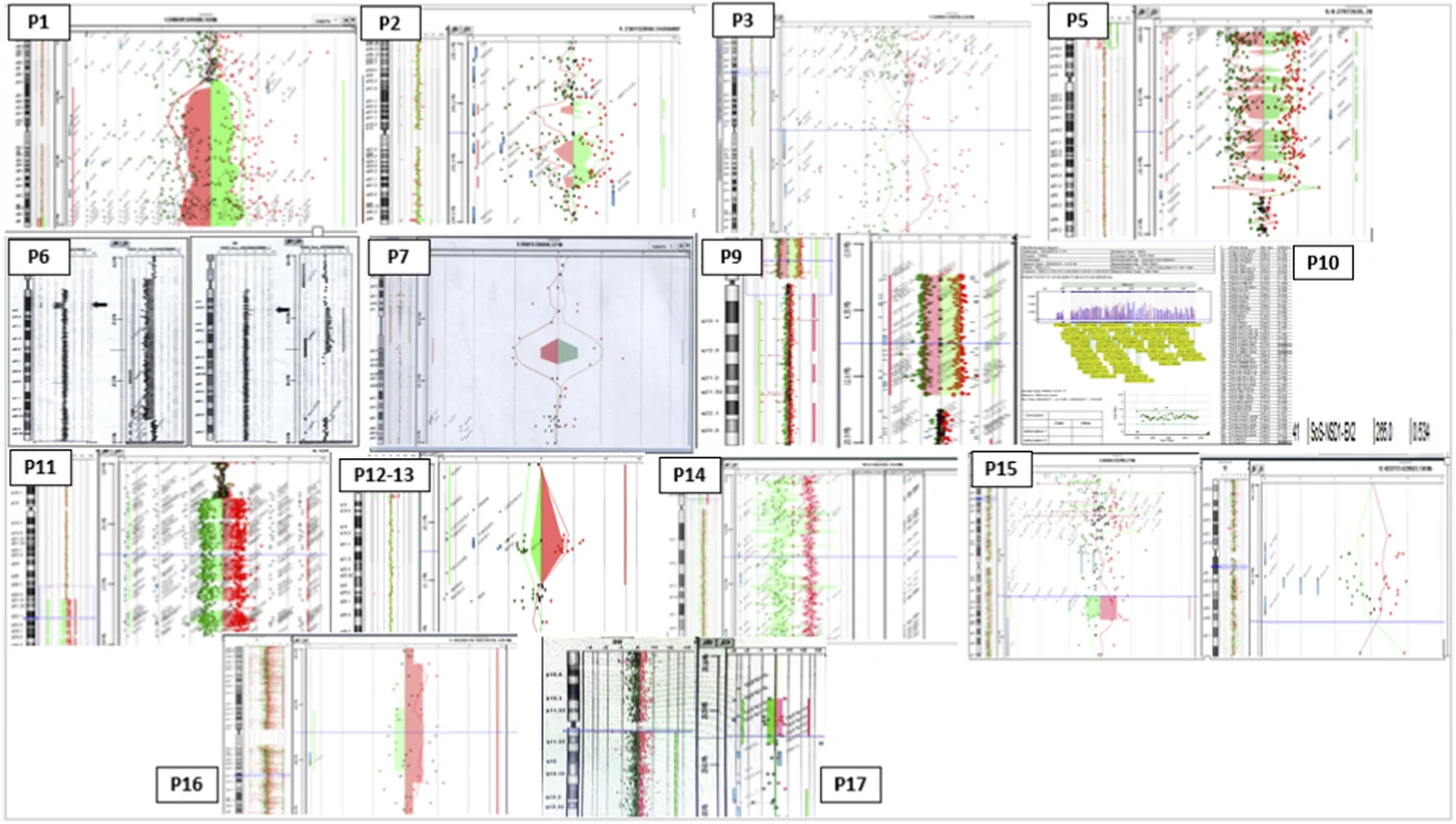
Array CGH results showing CNV detected in patients with CCM. P1: pathogenic loss of 11,7 Mb at 1q43q44, P2: pathogenic loss of 2.7 Mb at 1q43q44, P3: pathogenic interstitial loss at 1p32.3p31.3 of 8.69 Mb, P5: pathogenic loss at 5p14.3 of 21Mb, P6: double CNV: pathogenic loss at 14q12 of 3.5 Mb and a gain VUS at 15q13.3 of 350 kb, P7: VUS loss at 9p21.1 of 400 kb, P9: Pathogenic loss at 18pterp11.1 of 14,7 Mb, P10: MLPA profile of the P064 kit showing a DQ of 0.534 for probe N°41 corresponding to the deletion of exon 2 of the NSD1 gene, P11: Pathogenic gain at 4q31.21 of 45 Mb, P12-P13: VUS gain at 5q11.2 of 300.651 Kb, P14: pathogenic 18p11.32p11.21 gain (quadriplication) of 14.73 Mb, P15: double CNV: VUS gain at 2p23.2 of 410 Kb and VUS gain at 12q12 of 170 kb, P16: Gain VUS at 1q31.1 of 1.8 Mb, P17: Gain VUS at 20q11.22 of 149.4 Kb.

Pathogenic or likely pathogenic CNVs included losses involving 14q12, 4p16.3–p16.1, 1q43–q44, 1p32.3p31.3, 5p14.3, 16q23.1–q24.3, and 18pterp11.1, as well as gains involving chromosome 4q and an isochromosome 18p. These genomic regions have previously been implicated in corpus callosum abnormalities and other neurodevelopmental disorders. Variants of uncertain significance included gains in 15q11.2, 1q31.1, of 20q11.2, and 12q12. Patients carrying pathogenic or likely pathogenic CNVs generally presented with syndromic phenotypes involving developmental impairment and additional neurological and/or extracerebral manifestations consistent with the known clinical spectrum of the corresponding genomic disorders. However, the limited number of molecularly diagnosed cases and phenotypic heterogeneity precluded systematic genotype–phenotype correlation analyses.

### 3.4 Whole-exome sequencing

Whole-exome sequencing was performed in six selected patients whose clinical presentation remained incompletely explained after cytogenetic investigations. Rare candidate sequence variants were identified in four patients, whereas no clinically relevant sequence variants were detected in the remaining two patients. Detected variants are presented in Table 1.

Patient P7 carried a heterozygous missense variant in *MID1*, consistent with the X-linked localization of this gene in association with a 9p21 loss. Patient P16 harbored two missense variants in addition to gain VUS at 1q31; compound heterozygous variants in WLS, a gene associated with Zaki syndrome; however, the variants could not be classified as pathogenic according to ACMG/AMP criteria.; Patient P16 also carried a homozygous missense variant in *ERCC8*, classified as VUS according to ACMG/AMP criteria. Patients P17 and P18, two affected siblings presenting a shared phenotype, both carried the same homozygous missense variant in *VPS39* co-occurring with a 20q11.2 gain in one sibling. All identified sequence variants were confirmed by Sanger sequencing. According to ACMG/AMP criteria, all sequence variants remained classified as variants of uncertain significance. For homozygous variants identified in patients carrying CNVs, allele balance was assessed from sequencing read counts. The *ERCC8* c.598T>A (p.Tyr200Asn) variant in P16 showed an allele balance of 1.00 (67/67 alternate reads), while the *VPS39* c.1648G>T (p.Val550Leu) variant identified in P17 and P18 showed an allele balance of 0.99 (143/144 alternate reads), supporting true homozygous states. Informations about quality metrics are presented in Table 2.

**TABLE 2:** Sequencing quality metrics for homozygous candidate variants identified by WES.

Patient	Gene	Variant	Genotype	Total depth (DP)	REF reads	ALT reads	Allele balance
P16	ERCC8	c.598T>A p.(Tyr200Asn)	1/1	67	0	67	1.00
P17	VPS39	c.1648G>T p.(Val550Leu)	1/1	144	1	143	0.99
P18	VPS39	c.1648G>T p.(Val550Leu)	1/1	144	1	143	0.99

In several patients, sequence variants and CNVs co-occurred, including cases where both alterations were classified as VUS. This co-occurrence may suggest a potential contribution of oligogenic or modifier effects; however, the current data do not allow establishment of a definitive multilocus mechanism. Genes contained within pathogenic and VUS CNVs are detailed in Table 3. All sequence and structural variants identified in this study have been submitted to the ClinVar database, and the corresponding ClinVar submission accessions are provided in Tables 1, 3.

**TABLE 3:** Clinical and genomic characteristics of patients with pathogenic, likely pathogenic and selected VUS findings.

Patients	Sex/Age	CCM	Phenotype category (neurodevelopmental disorders/cerebral/extra-cerebral malformations	Karyotype/FISH/MLPA	Array CGH (ISCN 2020; hg18)	CNV (G/L)	Size	Genes of interest	Inheritance	ACMG/AMP	Clinvar accession	Clinical interpretation
P1	M 3 Y	T ACC	Psychomotor retardation behavioral disorders Axial hypotonia Encephalic malformation Microcephaly FD, hypospadias, ASD, moderate PS	46,XY, ish del(1)(q42q44)(RP11-156F12x1)	arr1q42q44(235,500,506–247,179,291)x1 dn	L	11,7 Mb	*ZBTB18* *PLD5* *FMN2*	*De novo*	P	SCV007595999	Consistent with distal 1q43q44 microdeletion syndrome
P2	M 9 M	HCC	Psychomotor retardation stature posterior delay Axial hypotonia pyramidal syndrome Decreased volume of the periventricular white matter Micropenis FD	46,XY, ish del(1)(q43q44)(RP11-99F4x1)	arr1q43q44(240,470,722–243,198,779)x1 dn	L	2.7 Mb	*ZBTB18* *PLD5* *FMN2*	*De novo*	P	SCV007596000	Consistent with distal 1q43q44 microdeletion syndrome
P3	M 4 Y	P ACC	Psychomotor retardation Speech delay Psychomotor agitation Autistic features Chiari type malformation Bifrontal atrophy localized pachygyria septum pellucidum cyst pineal gland cyst Minor macrocrania Clinodactyly of the 5th finger Micropenis	46,XY, ish del(1) (RP11-714D6x1)	arr1p32.3p31.3 (52,909,857–61,607,083)x1 dn	L	8.67 Mb	*PPAP2B* *DAB1* *HOOK1* *NFIA* *TMEM29* *BSND*	*De novo*	P	SCV007596001	*NFIA* deletion associated with corpus callosum abnormalities
P4	F 4 Y	HCC	Psychomotor retardation Absence of language Epilepsy FD ASD PS	46,XX, ish del (4p16.3) (WHSx1)	arr4p16.3(68,345–6,427,504)x1	L	6.36 Mb	*PIGG* *FGFRL1 WHSC1* *CTBP1* *CRMP1*	*De novo*	P	SCV007596002	Wolf–Hirschhorn syndrome critical region
P5	M 3 Y	ACC	Psychomotor retardation weight delay Partial agenesis of the vermis FD Atrophy of the testicles Single palmar fold on the left	46,XY,del(5)(p14)	Arr5p14.3(148,243–21,305,214)x1	L	21 Mb	*CDH 18* *CTNND2* *SEMA5A*	*De novo*	P	SCV007596003	Consistent with a cri-du-chat–spectrum cerebral phenotype (ACC, vermis hypoplasia)
P6	F 6 M	HCC	Psychomotor retardation Microcephaly discrete FD	46,XX, ish del(14q12)(RP11-120I18x1).ish dup(15q13.1)(RP11-680F8x3)	arr14q12(26,838,442–30,367,571)x1,arr15q13.3 (29,885,962–30,214,102)x3	14q12 (3.5 Mb) 15q13.3 (350 Kb)	L G	*14q12* *FOXG1* *PRKD1* *15q13.3: CHRNA7*	*De novo*	P V	SCV007596004/SCV007596005	14q12 deletion involving *FOXG1* consistent with FOXG1 syndrome; concurrent 15q13.3 gain involving *CHRNA7* of uncertain additional contribution
P7	F 11 Y	T ACC	Psychomotor retardation Weight delay Mental retardation FD	46,XX	Arr9p21(31,013,099–31,463,899)x1	450,8 Kb	L	*-*	*De novo*	V	SCV007596006	9p21.3 loss of uncertain significance, no candidate gene identified in the interval
P8	M 1 M	Dysgenesis of CC	Hydrocephaly Microcephaly	46,XY	Arr16q(74,718,513 87,891,103)x1	L	L	*WWOX* *ANKRD11* *ZNF778* *CDH15* *CDH13*	*De novo*	P	SCV007596007	Consistent with 16q23q24 microdeletion syndrome, including corpus callosum dysgenesis
P9	M 5 Y	T ACC	FD	46,XY, ish(18)(p11.31)(RP11-325E18x2)x1	arr18pterp11.1(170,229–14918854)X1	14,7 Mb	L	*TGIF1* *LAMA1* *DLGAP1*	*De novo*	P	SCV007596008	Consistent with 18p deletion syndrome and associated corpus callosum agenesis
P10	F 2 Y	HCC	Stature-weight gain Psychomotor retardation Septum pellucidum cysts Swelling of the anterior pituitary gland FD	46,XX Deletion of exon 2 of NSD1 gene 5q35.3	-	Deletion of exon 2 of NSD1 gene	L	*NSD1*	*De novo*	P	SCV007601128	Sotos syndrome region
P11	M 1 Y	ACC	Epilepsy Microcephaly FD Pulmonary narrowing Choanal atresia Anal imperforation Operated testicular ectopy Bilateral club feet	46,XY.dup4q(31.3), ish dup(4)(q31q35)(tel 4qx3)	Arr4q31.21(142,896,377–188,839,494)x3	45 Mb	G	*SPOCK3* *SMAD1* *DCLK2*	*De novo*	P	SCV007596009	Consistent with the epileptic phenotype and pulmonary valve stenosis observed
P12	M 6 Y	HCC	Psychomotor retardation Epilepsy with flexion spasms Behavioral disorders (manual stereotypy) language limited to a few syllables Hypoplasia of the vermis Left temporal atrophy Occipital atrophy Microcephaly, FD	46,XY	Arr15q11.2(20,335,887–20,636,537)x3	300.651 Kb	G	*NIPA1* *NIPA2* *CYFIP1*	*De novo*	V	SCV007596010	A region of variable, incompletely penetrant association with neurodevelopmental disorders
P13	F 1 Y	T ACC	Partial agenesis of the vermis FD	46,XX	Arr15q11.2(20,335,887–20,636,537)x3	300.651 Kb	G	*NIPA1* *NIPA2* *CYFIP1*	*De novo*	V	SCV007596010	A region of variable, incompletely penetrant association with neurodevelopmental disorders
P14	F 3 M	HCC	Microcephaly Single transverse palmar fold FD	47,xx + mar, ish der(18) (WCP18x3) dn	Arr18p (198,111–14,928,854)x4	14,73 Mb	G Isochromosome 18p	*DLGAP1* *TGIF1* *PIEZO2* *LAMA1*	*De novo*	P	SCV007596011	Consistent with tetrasomy 18p syndrome
P15	M 1 M	HCC	Gyration abnormality Microcephaly FD	46,XY	arr2p23.2(28,837,963–29,251,002)x3 12q12(40,840,168–41,015,988)x3	2p23.2 (410 Kb) 12q12 (170 Kb)	G	*2p23.2* *PPP1CB* *12q12* *YAF2* *ZCRB1* *PPHLN1*	*De novo*	2p23.3 V 12q12 V	SCV007596012/SCV007596013	Uncertain significance and unclear individual contribution to the phenotype
P16	F 7 Y	HCC	Weight delay Psychomotor regression at 9 months and regression of language acquisition at 1 year Spastic paraplegia Hypomyelination of the white matter FD Bilateral V clinodactyly Microcephaly Thoracic kyphosis Multiple warts	46,XX	Arr1q31.1(187,053,276–188,891,105) x3	1.8 Mb	G	*BRINP3*	*De novo*	V	SCV007596014	Uncertain significance with possible contribution to the neurodevelopmental regression phenotype
P17	M 1Y	HCC	Right hemiparesis Left subcortical atrophy	46,XY	Arr20q11.22(29,352,138–29,501,594)x3	149,4 Kb	G	*DEFB118*	*De novo*	V	SCV007596015	Uncertain significance, no candidate gene identified in the interval

Abbreviations: ACC: agenesis of corpus callosum, ASD: atrial septal defect, CC: Corpus Callosum, CCM: corpus callosum malformations, dn:de novo, FD: facial Dysmorphia, HCC: hypoplasia of corpus callosum, F: Female, G: Gain, M: Months, M: Male, L: Loss, P: pathogen, PS: pulmonary stenosis, V: VUS, +: explored by WES, -: not explored by WES, Y: years.

## 4 Overall diagnostic yield

Overall, clinically relevant genomic abnormalities were identified in 11 of 107 patients (10.3%). Conventional karyotyping identified chromosomal abnormalities in three patients (2.8%), while array-CGH detected pathogenic or likely pathogenic CNVs in ten of the 54 tested patients (18.5%). Whole-exome sequencing, performed only in a highly selected subgroup of unresolved patients, identified rare candidate sequence variants in four patients; however, all sequence variants remained classified as variants of uncertain significance and therefore were not included in the overall diagnostic yield. Detailed informations are presented in Table 4.

**TABLE 4:** Diagnostic yield of cytogenetic and genomic testing in the study cohort.

Diagnostic level	Method	Patients tested (n/N)	Positive findings	Diagnostic yield	Comments
Conventional cytogenetics	Karyotyping	107/107	3 abnormal karyotypes	2.8%	Structural chromosomal abnormalities
Molecular cytogenetics	FISH	24/107	7 confirmed cases (confirmation rate)	29.2% of tested patients	Primarily used for confirmation and characterization of chromosomal abnormalities
CNV analysis	Array-CGH	54/107	18 CNVs detected in 16 patients	29.6% of tested patients	Includes pathogenic, likely pathogenic, and VUS CNVs
Clinically relevant CNVs	Array-CGH	54/107	10 pathogenic/likely pathogenic CNVs in 10 patients	18.5% (10/54)	Diagnostic yield based on ACMG/ClinGen classification
Targeted CNV analysis	MLPA	One patient (P10)	1 pathogenic *NSD1* exon 2 deletion	Not calculated	Targeted analysis in P10
Sequence analysis	Whole-exome sequencing (WES)	6/107	5 rare VUS variants identified in 4 patients	Not calculated*	Applied only to selected unresolved cases with CNVs and/or consanguinity

* WES, was applied only to highly selected unresolved cases and yield is not directly comparable.

## 5 Discussion

Corpus callosum abnormalities (CCA) represent a heterogeneous group of neurodevelopmental disorders resulting from disturbances in midline patterning, neuronal migration, axonal guidance, and commissural development. Their clinical spectrum ranges from isolated callosal defects to severe syndromic presentations associated with intellectual disability, epilepsy, dysmorphic features, and additional brain or systemic abnormalities. Despite advances in genomic technologies, establishing genotype–phenotype correlations remains challenging due to the large number of genes and genomic regions involved.

### 5.1 Diagnostic contribution of a stepwise genomic strategy in corpus callosum abnormalities

In this study, we investigated 107 Tunisian patients with radiologically confirmed CCA using a stepwise genomic approach integrating conventional karyotyping, fluorescence *in situ* hybridization (FISH), Multiplex Ligation-dependent Probe Amplification (MLPA), chromosomal microarray analysis (array-CGH), and whole-exome sequencing (WES) in selected unresolved cases. The cohort illustrates the marked phenotypic and genomic heterogeneity of CCA and highlights the complementary contribution of different genomic approaches for molecular investigation in patients with developmental disorders and structural brain abnormalities.

Using this sequential diagnostic strategy, clinically relevant genomic abnormalities were identified in 11 of the 107 patients (10.3%). Conventional karyotyping contributed to the diagnosis of three patients (2.8%), while Array-CGH performed in 54 selected patients identified 18 CNVs in 16 individuals (29.6%), including pathogenic or likely pathogenic CNVs in 10 patients (18.5%). The pathogenic or likely pathogenic CNVs identified by array-CGH involved 10 patients (P1, P2, P3, P4, P5, P6, P8, P9, P11, and P14), whereas the remaining CNVs were classified as variants of uncertain significance according to ACMG/ClinGen recommendations.

Whole-exome sequencing was performed in six selected unresolved cases and identified rare sequence variants in four patients; however, all variants remained classified as variants of uncertain significance (VUS) according to ACMG/AMP criteria and were therefore not included in the diagnostic yield.

The overall diagnostic yield observed in our cohort should be interpreted according to the applied testing strategy. Unlike prospective studies in which patients undergo systematic chromosomal microarray followed by sequencing, our cohort was investigated over a 15-year period during which access to genomic technologies evolved progressively. Array-CGH was not performed in all patients, and WES was restricted to selected unresolved cases rather than systematically applied after negative chromosomal microarray results. Consequently, the proportion of patients with an identifiable genetic etiology may be underestimated. The observed yield therefore reflects the contribution of available diagnostic approaches in a real-world tertiary-care setting rather than the complete genetic burden of CCA.

### 5.2 Clinical significance of pathogenic and likely pathogenic copy-number variations

Chromosomal microarray analysis has contributed significantly to the identification of pathogenic copy-number variations (CNVs) in neurodevelopmental disorders, including CCA. Since early large-scale studies highlighting critical genomic regions (O’Driscoll et al., 2010), stepwise approaches combining cytogenetics with next-generation sequencing (NGS) have refined the understanding of CCA genetics.

Copy-number variations represented the main category of clinically relevant genomic abnormalities identified in our cohort. The pathogenic and likely pathogenic CNVs detected by array-CGH involved chromosomal regions that have previously been implicated in neurodevelopmental disorders and corpus callosum development, including deletions affecting 14q12, 4p16.3–p16.1, 1q43–q44, 1p32.3p31.3 –p32, 5p14.3, 5q35, 16q23.1–q24.3, and 18pterp11.1, as well as gains involving chromosome 4q and an isochromosome 18p. These findings reinforce the contribution of genomic dosage imbalance to the pathogenesis of CCA and support the existence of recurrent genomic mechanisms affecting commissural development.

Our findings expand previous Tunisian reports that primarily focused on individual chromosomal abnormalities or rare genetic syndromes associated with corpus callosum anomalies (Boukhris et al., 2008; Ouertani et al., 2009; Khadija et al., 2022; Kerkeni et al., 2026). In contrast to earlier case reports and small series, the present study integrates clinical, cytogenetic, CNV, and sequencing data from a larger cohort of 107 patients, providing a broader overview of the genetic landscape of CCA in Tunisia.

These recurrent regions are consistent with prior international CCA cohorts. In the largest comparable series, chromosomal microarray in 149 patients with corpus callosum abnormality and intellectual disability identified pathogenic CNVs in 13% of cases, with recurrent involvement of 1q43–q44, 14q12, and 18q21 among others (Depienne et al., 2017) a yield closely comparable to the 16.7% observed in our array-CGH subgroup. Similarly, the comprehensive genomic-loci map constructed by (Sajan et al., 2013) from 374 ACC patients, and the case-control CNV discovery study of (Sajan et al., 2013), encompassing 255 ACC patients, both support 1q43–q44 and 4p16.3 (Wolf–Hirschhorn syndrome) as recurrent, high-penetrance loci for callosal malformation, consistent with our findings in P1–P4. Our identification of a 14q12 deletion spanning *FOXG1* similarly aligns with dedicated case series delineating this region as a critical locus for corpus callosum hypoplasia.

Among the identified abnormalities in our study, deletions involving 1q43–q44 represent a well-established genomic region associated with severe neurodevelopmental phenotypes including corpus callosum abnormalities. In our cohort, two patients carried deletions involving this region (P1, P2). The 1q43–q44 interval contains several genes implicated in neuronal development, including ZBTB18 (OMIM *608433), a transcriptional regulator involved in neuronal differentiation, cortical development, and brain patterning. Previous studies have demonstrated that haploinsufficiency of *ZBTB18* is associated with developmental delay, epilepsy, brain malformations, and abnormal corpus callosum development. The identification of deletions affecting this region in our patients is therefore consistent with its established contribution to neurodevelopmental disorders (Khadija et al., 2022).

A large deletion involving 1p32.3p31.3 was identified in patient P3 and included NFIA (OMIM*600727), a major regulator of central nervous system development. *NFIA* plays important roles in glial differentiation, neuronal migration, and axonal development (Lu et al., 2007; Nyboe et al., 2015; Bayat et al., 2017). Pathogenic variants and deletions involving *NFIA* have been repeatedly associated with corpus callosum abnormalities, ventriculomegaly, developmental delay, and other structural brain anomalies (Schirwani Smith et al., 2018).

Similarly, deletion involving 4p16.3–p16.1 were identified in patient P4 presenting with corpus callosum abnormalities and multisystem manifestations. This region contains several dosage-sensitive genes, including FGFRL1 (OMIM*605830), CTBP1 (OMIM*602618) and *PIGG* (OMIM*616918), suggesting combined effects on transcriptional regulation and neuronal protein modification(Callaway et al., 2018; Sidpra et al., 2024). Alterations affecting *FGFRL1* have previously been associated with disorders involving forebrain development and midline abnormalities, supporting the pathogenic relevance of this genomic imbalance.

The deletion involving 14q12 in P6 contains the *FOXG1* gene (OMIM*164874), a critical regulator of forebrain development. *FOXG1* haploinsufficiency is associated with a severe neurodevelopmental disorder characterized by developmental impairment, epilepsy, microcephaly, and brain structural abnormalities, including corpus callosum defects. The concurrent loss of *PRKD1* (OMIM * 605,435), which may influence *FOXG1* expression, together with a 15q13.3 gain in the same patient P6 including *CHRNA7* (OMIM * 118,511), suggests the possibility of additional modifying effects (Oster, Abraham et Leitges, 2006; Takagi et al., 2013; Sinkus et al., 2015). However, their contribution to phenotypic severity cannot be established from the current data.

Other pathogenic CNVs identified in our cohort involved genes as LAMA1 (OMIM*150320), SPOCK3 (OMIM*607989), *DCLK2*, CTNND2 (OMIM*604275), SEMA5A (OMIM *609297), and *CDH18* (OMIM *603019) highlight the importance of neuronal positioning and connectivity in commissural development. Disruption of these genes has been associated with cortical malformations frequently co-occurring with CCA, such as polymicrogyria, heterotopia, and lissencephaly, and is often accompanied by developmental delay, epilepsy, and neuropsychiatric manifestations (Redies, Hertel et Hübner, 2012; Hartmann et al., 2013; Chen et al., 2013; Micalizzi et al., 2016). These findings emphasize that CCA may result from disruption of multiple genomic regions converging on common developmental processes, including neuronal migration, axonal connectivity, and brain morphogenesis.

In addition to pathogenic CNVs, several CNVs classified as variants of uncertain significance were identified, including recurrent gains involving 15q11.2, 1q31.1, and 2p23.2. The interpretation of these findings remains challenging because some genes such as *NIPA1* (OMIM*608145), *NIPA2* (OMIM*608146), *CYFIP1* (OMIM*606322), *BRINP3* (OMIM*618390), *PPP1CB* (OMIM*600590) located within these regions have been associated with neurodevelopmental phenotypes, but current evidence is insufficient to establish a direct causal relationship with CCA.

For example, recurrent alterations involving the 15q11.2 region have been reported in association with variable neurodevelopmental manifestations, including developmental delay, intellectual disability, epilepsy, and behavioral disorders. Several studies has shown that 15q11.2 gains were identified in 0.42% of people referred for genetic testing (215 out of 51,462people (Mohan et al., 2019). However, the contribution of individual genes within this interval to corpus callosum abnormalities remains uncertain. Similarly, the 1q31.1 gain involving *BRINP3* represents an interesting candidate finding, but additional evidence is required before assigning a pathogenic role.

Several genomic alterations identified in our cohort affect genes involved in biological processes relevant to commissural development, including transcriptional regulation (*ZBTB18, FOXG1*, *NFIA*), neuronal migration (*LAMA1, DCLK2*), and axonal guidance mechanisms (*SEMA5A*). Although direct functional relationships between individual CNVs and corpus callosum formation cannot always be established, these findings suggest convergence of multiple developmental pathways underlying CCA.

Although several CNVs identified in this cohort correspond to established genomic disorders, their clinical expression is influenced by genomic content, genetic background, and possible modifying factors. Therefore, the presence of a pathogenic CNV does not imply a uniform phenotype, and genotype–phenotype interpretation requires consideration of individual clinical features and additional genomic findings. Therefore, while pathogenic CNVs identified in this cohort support established mechanisms of genomic imbalance in CCA, VUS CNVs should be interpreted cautiously and considered as candidate genomic findings requiring additional segregation studies, functional investigations, and identification of independent cases.

### 5.3 Interpretation of rare sequence variants and co-occurring genomic alterations

Rare sequence variants were identified in *MID1, WLS, ERCC8, and VPS39*; however, all remained classified as variants of uncertain significance (VUS) according to ACMG/AMP criteria, only in *MID1* classified as likely benign. In one patient, compound heterozygous variants in *WLS* (c.985C>T, p.(Arg329Trp) and c.1124C>A, p.(Thr375Lys)) were identified and classified as VUS (PM2, PP3) based on their rarity in population databases and computational predictions suggesting a potential functional impact. This patient also carried a 1q31.1 gain classified as VUS, involving *BRINP3*, a gene previously reported in neurodevelopmental disorders. Although biallelic pathogenic variants in WLS are associated with Zaki syndrome and *BRINP3* represents a biologically plausible candidate gene, the available evidence remains insufficient to establish a causal contribution of either finding to the observed phenotype.

The same patient also harbored a homozygous *ERCC8* variant (c.598T>A, p. Tyr200Asn), classified as a variant of uncertain significance based on ACMG/AMP evidence (PM2, PM1). Although *ERCC8* is associated with Cockayne syndrome and the patient displayed dysmorphic features suggestive of this condition, other characteristic manifestations such as photosensitivity, pigmentary retinopathy, sensorineural hearing loss were not reported. Therefore, the available clinical and molecular data remain insufficient to establish a diagnosis of Cockayne syndrome.

A homozygous missense variant in *VPS39* (c.1648G>T, p. Val550Leu), identified in two affected siblings (P17-P18), was classified as a variant of uncertain significance (PM2, PP3). *VPS39* encodes a core component of the HOPS complex, which regulates late endosomal and lysosomal fusion, autophagy, and intracellular vesicle trafficking. The p.(Val550Leu) substitution is located within the C-terminal region of the protein, involved in HOPS complex assembly. Although the affected residue is predicted to be functionally relevant and disruption of endolysosomal trafficking represents a biologically plausible mechanism for neurodevelopmental disorders affecting neuronal connectivity and white matter development, there is currently insufficient evidence to establish this variant as disease-causing. One sibling (P17) additionally carried a 20q11.2 gain classified as a VUS; however, because both siblings shared a highly similar clinical phenotype despite the duplication being present in only one of them, this CNV is unlikely to explain the shared phenotype or to represent a major phenotypic modifier. Furthermore, VPS39 is not currently an established disease gene for isolated corpus callosum abnormalities according to available OMIM and GenCC evidence. Therefore, the variant remains classified as a VUS pending additional functional and segregation studies.

Finally, a heterozygous *MID1* missense variant (c.250G>A, p. Asp84Asn) was identified in one patient but was classified as likely benign according to ACMG/AMP criteria (PM2, BP6, BS2). Accordingly, this variant was not considered to contribute to the patient’s phenotype. The accompanying 9p21 deletion also remained a variant of uncertain significance, and neither finding established a molecular diagnosis.

Several patients investigated by WES presented co-occurring CNVs and rare sequence variants. However, because WES was performed only in selected unresolved patients with complex phenotypes, consanguinity, and/or previously identified CNVs, these observations should not be interpreted as evidence of oligogenic inheritance. Rather, they represent hypothesis-generating findings requiring additional segregation studies, functional validation, and identification of unrelated cases to determine whether multiple genomic alterations contribute to phenotypic variability in a subset of corpus callosum abnormalities.

The identification of variants in biologically relevant genes does not establish causality in the absence of sufficient ACMG evidence, segregation data, or functional validation.

### 5.4 Biological interpretation and clinical implications

Network analyses using STRING (https://string-db.org/) and GeneMANIA (https://genemania.org/) (Figures 7A,B) highlighted biological processes related to neuronal development, axonal guidance, and brain morphogenesis. However, these analyses provide functional context rather than independent evidence of pathogenicity and should therefore be considered exploratory.

**FIGURE 7:**
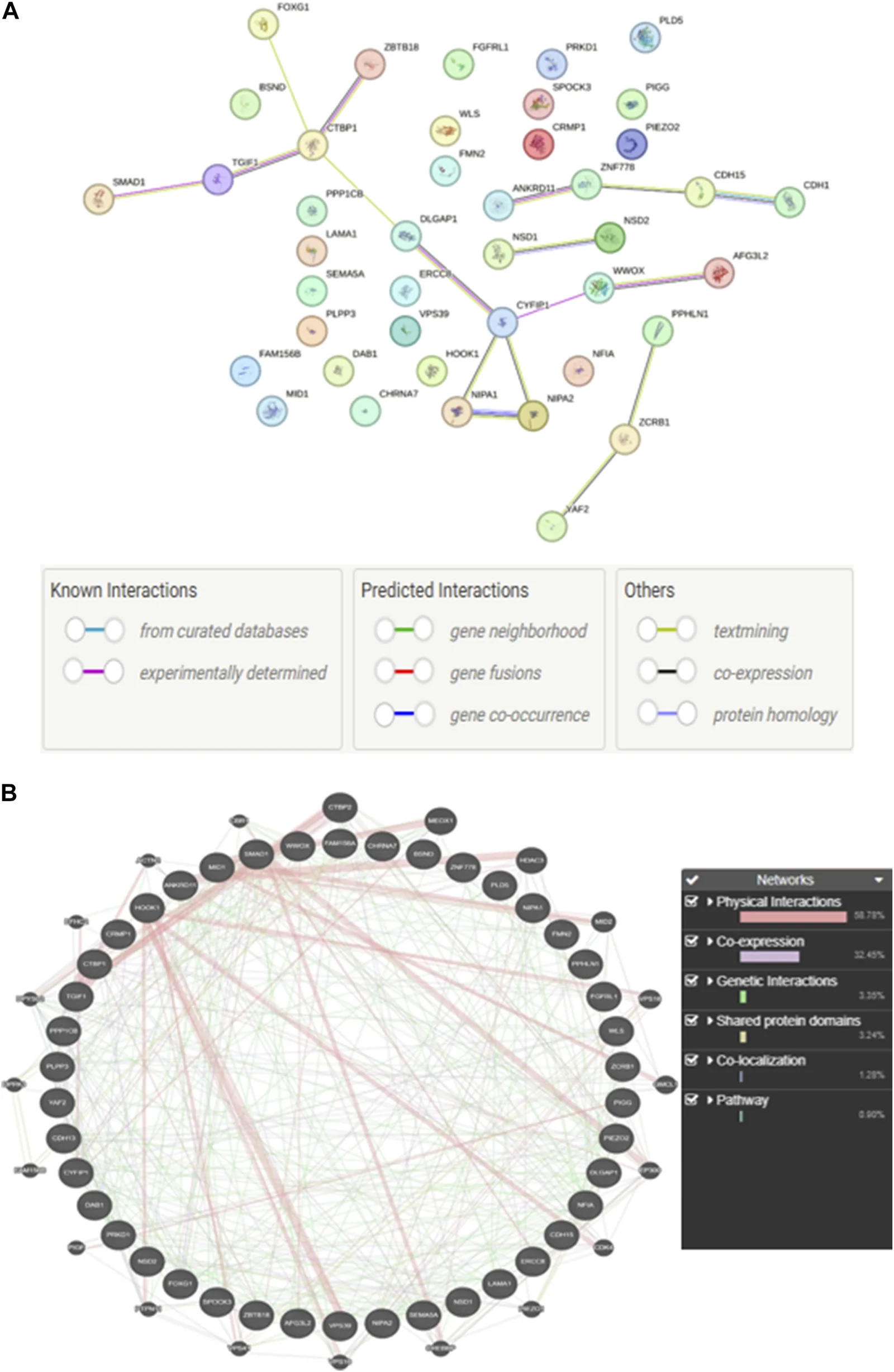
**(A)** Gene interaction using STRING data base. **(B)** gene interaction using GENEMANIA data base. This network illustrates predicted functional and physical interactions among the genes identified in our CCM cohort.

From a clinical perspective, our results support the value of integrating complementary genomic approaches in CCA. Array-CGH remains an important diagnostic tool for identifying pathogenic CNVs, particularly in syndromic cases, while sequencing approaches may help identify candidate variants in unresolved patients. However, careful interpretation according to ACMG criteria remains essential, particularly when rare variants lack functional or segregation evidence.

### 5.5 Strengths and limitations

This study represents one of the largest clinically characterized Tunisian cohorts of patients with CCA investigated using complementary genomic approaches. The integration of cytogenetics, array-CGH, and WES provides valuable information regarding the genomic heterogeneity of CCA in an underrepresented population. Furthermore, variant interpretation according to ACMG/AMP and ACMG/ClinGen recommendations and submission of variants to ClinVar improve data transparency and future reinterpretation.

Several limitations should be acknowledged. The retrospective hospital-based design introduces referral bias and prevents epidemiological conclusions. Genomic investigations were not uniformly performed across all patients, with WES restricted to six selected cases. The recruitment period (2010–2025) also encompassed substantial technological evolution, potentially influencing detection sensitivity. In addition, parental segregation studies were incomplete, functional validation was unavailable, and analyses such as systematic runs of homozygosity assessment were beyond the scope of this study.

### 5.6 Clinical implications and future perspectives

From a clinical perspective, our findings support the value of a stepwise genomic strategy for patients with corpus callosum abnormalities, particularly in individuals presenting with syndromic features, developmental impairment, dysmorphic signs, or additional brain malformations.

Chromosomal microarray analysis remains an important diagnostic tool because pathogenic CNVs represent a significant proportion of clinically relevant findings in CCA. Conventional karyotyping also retains value for detecting large chromosomal abnormalities, while sequencing approaches provide additional opportunities for investigating unresolved cases.

However, the interpretation of sequence variants remains a major challenge, particularly when multiple rare findings are identified in patients with complex phenotypes. The implementation of comprehensive genomic strategies should therefore be accompanied by careful variant classification, parental studies whenever possible, and functional validation approaches.

Future studies involving larger multicenter cohorts, systematic application of genome-wide sequencing, complete segregation analyses, functional investigations, and international data sharing will be essential to refine genotype–phenotype correlations and improve molecular diagnosis in corpus callosum abnormalities.

## 6 Conclusion

In conclusion, this study provides a comprehensive clinical and genomic characterization of corpus callosum abnormalities in a Tunisian referral cohort. The integration of conventional cytogenetics, chromosomal microarray analysis, and targeted whole-exome sequencing identified clinically relevant genomic abnormalities while highlighting the persistent challenges associated with interpretation of rare genomic variants.

Pathogenic and likely pathogenic CNVs represented the main diagnostic findings, supporting the major contribution of genomic dosage imbalance to corpus callosum development. Conversely, rare sequence variants identified by WES remained classified as variants of uncertain significance and require additional evidence before their clinical relevance can be established.

Overall, our findings emphasize the genetic heterogeneity of corpus callosum abnormalities and the complementary role of multiple genomic approaches in their investigation. Larger collaborative studies incorporating systematic genomic testing, segregation analyses, and functional validation will be required to further improve molecular diagnosis and refine genotype–phenotype relationships in these complex neurodevelopmental disorders.

## Data Availability

All sequence variants reported in this study have been submitted to the ClinVar database and are available under the corresponding ClinVar accession numbers listed in Table 1. Copy-number variations (CNVs) were interpreted according to ACMG/ClinGen guidelines and their ClinVar accession numbers are listed in Table 3. Corresponding ClinVar Variation IDs (VCV) will be added upon release. Additional data supporting the findings of this study are available from the corresponding author upon reasonable request.

## Glossary

Array CGH: Array comparative genomic hybridization
ACMG: American College of Medical Genetics and Genomics
*AKT3*: AKT SERINE/THREONINE KINASE 3
*ANKRD11*: ankyrin repeat-containing cofactor 1
*AMPD2*: adenosine monophosphate deaminase
*BRINP3*: BMP and Retinoic Acid Induced Neuro-specific Protein three
BMP4: Bone Morphogenetic Protein
CCA: Corpus Callosum Abnormalities
CCM: Corpus Callosum Malformations
CC: Corpus Callosum
*CEP170*: Centrosomal Protein 170
*CTBP*1: c-terminal-binding protein one
*CHRNA7*: cholinergic receptor, neuronal nicotinic, alpha polypeptide 7
*CYFIP1*: cytoplasmic fmrp-interacting protein one
*CWC27*: spliceosome-associated cyclophilin
*CDH13/18*: cadherin 13/18
DAPI: Diamino-2-phenylindole
DNA: Desoxyribonucleic acid
*DHCR7*: 7-DEHYDROCHOLESTEROL REDUCTASE
*DCLK2*: Doublecortin-Like Kinase 2
Draxin: DORSAL INHIBITORY AXON GUIDANCE PROTEIN
FISH: Fluorescence *in situ* hybridization;
*FMN2*: Formin 2
*FGFR1*: Fibroblast Growth Factor Receptor one
*FGFR2*: fibroblast growth factor receptor 2
*FOXG1*: FORKHEAD BOX G1
HCC: Hypoplasia of the Corpus Callosum
*HNRNPU*: Heterogeneous Nuclear Ribonucleoprotein U
ISCN: International System for Human Cytogenetic Nomenclature 2020
MLPA: Multiplex Ligation-dependent Probe Amplification
OMIM: Online Mendelian Inheritance in man
*PLD5*: Phospholipase D Family Member 5
*RGS7*: Regulator Of G Protein Signaling 7
*SMYD3*: SET And MYND Domain Containing 3
*SDCCAG8*: SHH Signaling And Ciliogenesis Regulator SDCCAG8
VUS: Variant Of Uncertain Significance
*VPS39*: VACUOLAR PROTEIN SORTING 39
*WLS*: WNT LIGAND SECRETION MEDIATOR
*Wnt5a*: WINGLESS-TYPE MMTV INTEGRATION SITE FAMILY, MEMBER 5A
*WDR43*: WD REPEAT-CONTAINING PROTEIN 43
*WWOX*: WW DOMAIN-CONTAINING OXIDOREDUCTASE
*YAF2*: YY1-ASSOCIATED FACTOR 2
*ZCRB1*: ZINC FINGER CCHC DOMAIN- AND RNA-BINDING MOTIF-CONTAINING PROTEIN 1
ZBTB18: ZINC FINGER- AND BTB DOMAIN-CONTAINING PROTEIN 18 (
