# Supplementary material for: Clinical and genomic characterization of corpus callosum abnormalities (CCA) in 107 Tunisian patients using a stepwise diagnostic approach
Source: Front Genet. 2026 Sep 14;17:1815268. doi: 10.3389/fgene.2026.1815268 (PMC13617407; doi:10.3389/fgene.2026.1815268)
Supplement: Supplementary file 1 [file Supplementaryfile1.docx]

# STROBE Checklist for Cross-sectional Observational Studies

**Manuscript: Clinical and Genomic Characterization of Corpus Callosum Abnormalities (CCA) in 107 Tunisian Patients Using a Stepwise Diagnostic Approach**

| Item | STROBE recommendation | Reported in manuscript | Section |
| --- | --- | --- | --- |
| 1 | Title/abstract indicate study design | Cross-sectional observational study stated in Methods; structured abstract. | Abstract, Methods |
| 2 | Background/rationale | Introduction | Introduction |
| 3 | Objectives | End of Introduction | introduction |
| 4 | Study design | Methods: Patient selection | Materials and Methods |
| 5 | Setting | Department of Genetics, 2010–2025 | Materials and Methods |
| 6 | Participants | Eligibility and recruitment described | Materials and Methods |
| 7 | Variables | Clinical, MRI and genomic variables defined | Materials and Methods |
| 8 | Data sources/measurement | Clinical, MRI, karyotype, FISH, array-CGH, MLPA, WES | Materials and Methods |
| 9 | Study size | 107 patients included | Materials and Methods |
| 10 | Quantitative variables | Diagnostic yields and proportions reported | Results |
| 11 | Descriptive data | Clinical characteristics summarized | Results |
| 12 | Outcome data | Genetic findings reported | Results |
| 13 | Main results | Diagnostic yields by platform | Results |
| 14 | Other analyses | exploratory genotype-phenotype discussion | Discussion |
| 15 | Key results | Discussion opening | Discussion |
| 16 | Limitations | Dedicated limitations section | Discussion |
| 17 | Interpretation | Discussion | Discussion |
| 18 | Generalisability | Clinical implications/future perspectives | Conclusion |
